# Dual energy CT and deep learning for an automated volumetric segmentation of the major intracranial tissues: Feasibility and initial findings

**DOI:** 10.1002/mp.70217

**Published:** 2025-12-21

**Authors:** Veronica Fransson, Filip Winzell, Birgitta Ramgren, Søren Christensen, Kristina Ydström, Ida Arvidsson, Niels Christian Overgaard, Kalle Åström, Anders Heyden, Johan Wassélius

**Affiliations:** ^1^ Department of Hematology, Oncology and Radiation Physics Skåne University Hospital Lund Sweden; ^2^ Department of Translational Medicine Medical Radiation Physics, Lund University Malmö Sweden; ^3^ Computer Vision and Machine Learning, Centre for Mathematical Sciences Lund University Lund Sweden; ^4^ Department of Medical Imaging and Physiology Skåne University Hospital Lund Sweden; ^5^ Department of Clinical Sciences Lund University Lund Sweden; ^6^ GrayNumber Analytics, A/B Copenhagen Denmark; ^7^ Department of Medical Radiation Physics Lund University Lund Sweden

**Keywords:** Brain, Deep Learning, Tissue segmentation, X‐Ray Computed Tomography Scanner

## Abstract

**Background:**

Magnetic resonance imaging (MRI) has traditionally been preferred over computed tomography (CT) for segmentation of intracranial structures due to its superior low contrast resolution. However, a reliable CT‐based segmentation could improve patient management when MRI is not practical. Despite advancements in CT imaging, such as enhanced tissue differentiation using virtual monoenergetic imaging (VMI) from dual energy CT, volumetric analysis remains underexplored.

**Purpose:**

The aim was to evaluate the feasibility of using deep learning (DL) models for segmentation of gray matter (GM), white matter (WM), and cerebrospinal fluid (CSF)—using virtual monoenergetic images (VMI).

**Methods:**

The study included 26 patients (training/validation: 21, test: 5) who underwent brain imaging on a dual‐layer CT and a T1‐weighted MR scan. MR‐based segmentation of GM, WM, and CSF served as the ground truth for training and testing of the DL models. Models included a baseline U‐Net++ trained on 70 keV VMIs and several U‐Net and U‐Net++ extensions designed to leverage spectral information from multiple VMIs (50, 70, and 120 keV). Model performance was evaluated using Dice Similarity Coefficient (DSC) and volumetric accuracy.

**Results:**

The U‐Net++ (Aug) model, using VMIs as augmentations of the input data, outperformed the baseline and other models with DSC 0.84, 0.77, and 0.88 for WM, GM, and CSF, respectively. The superiority was significant compared to several of the other models, and most notably compared to the baseline model with DSC of 0.81 for WM (*p* = 0.002) and 0.75 for GM (*p* = 0.002). U‐Net++ (Aug) had an average volumetric error of 12%, while U‐Net (Gated) had the lowest error at 10%.

**Conclusions:**

This study demonstrates the feasibility of CT‐based segmentation of intracranial tissue using DL and VMI. The improved accuracy of the U‐Net++ (Aug) compared to the baseline model suggests that spectral information may enhance segmentation performance.

**Abbreviations**
AVD: Average Volumetric Difference
CI: Confidence Interval
CSF: Cerebrospinal Fluid
CT: Computed Tomography
CTDI_vol_
: Volumetric computed tomography dose index
DSC: Dice Score Coefficient
GM: Gray Matter
IQR: Inter‐Quartile Range
keV: kiloelectronvolt
MRI: Magnetic Resonance Imaging
PACS: Picture, Archiving and Communication System
VMI: Virtual Monoenergetic Image
WM: White Matter

## 1 INTRODUCTION

Computed tomography (CT), alongside magnetic resonance imaging (MRI), forms the backbone of neuroimaging. While MRI is often favored for its superior resolution in differentiating tissues with similar compositions—partly made possible by the many different sequences available—CT remains an essential tool in clinical practice due to its wide availability, speed of acquisition, and detailed imaging of bone structures. A reliable segmentation of brain tissues from CT would be valuable for diagnosing and monitoring disease progression or treatment effect in neurodegenerative and other diseases, especially in cases where MRI is not feasible.^1^ Despite this potential, CT‐based volumetry has seen limited use, primarily in specialized applications like brain perfusion imaging, hemorrhage volume measurement, and early ischemia assessment.^2^, ^3^ Contrary, there are numerous examples of volumetry—and the added clinical value thereof—using MRI.^4^, ^5^, ^6^, ^7^, ^8^ Advancements in CT technology have led to dual energy imaging and the possibility of generating virtual monoenergetic images (VMI), which can improve the differentiation between normal gray and white matter as well as between healthy and diseased gray matter.^9^, ^10^, ^11^, ^12^ VMIs also have lower noise levels compared to conventional, polyenergetic images. These technological improvements have improved the conditions for the development of deep learning models for segmenting intracranial tissues from CT.

**FIGURE 1:**
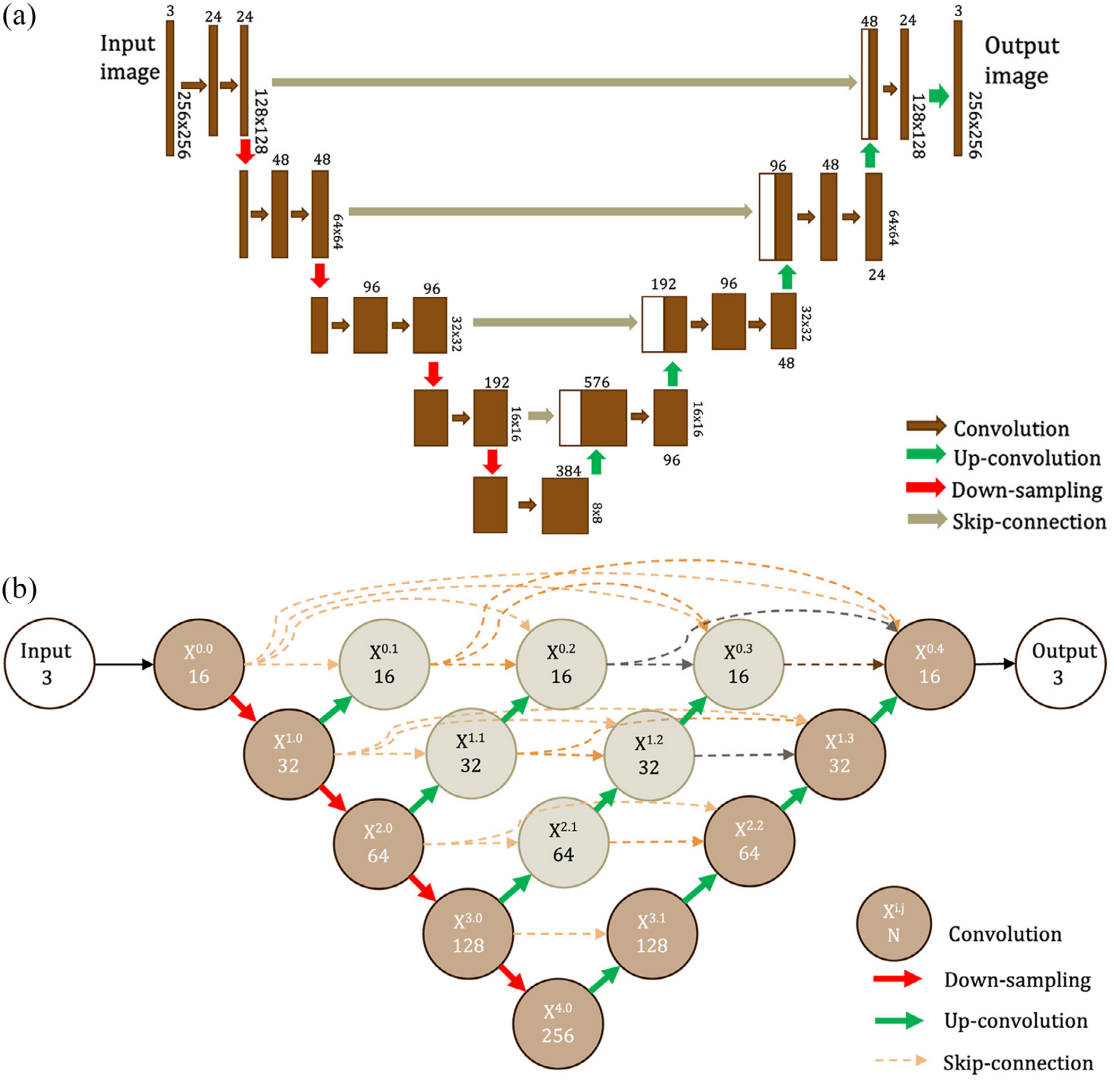
(a) The U‐Net architecture used in this study (3.5 M parameters). Numbers indicate the number of channels of the block, followed by a convolutional layer or a concatenation of a skip‐connection and up‐convolution. (b) The U‐Net++ architecture (2.6 M parameters) extends the U‐Net by adding several skip‐connections and up‐convolutions, creating a set of connected nested decoder sub‐networks. The number indicates the filters of the i,j‐th convolution block ($X^{i,j}$).

Traditional automated segmentation using CT number thresholds has been challenging due to the variability of CT numbers (densities) between cases, CT scanners, and imaging protocols. Over the past decade, deep learning (DL) models have gained significant research interest as they offer a potential solution to this problem by eliminating the need for predefined thresholds or features for segmenting.^13^, ^14^, ^15^, ^16^ The U‐Net architecture (Figure 1a), with its many variations, has become a popular choice in numerous studies.^17^ Its main advantage lies in enhancing the model's strength without adding more trainable parameters, which is useful in settings with limited training images.

U‐Net++ (Figure 1b), a popular extension of U‐Net has shown improved performance in CT image segmentation, particularly for tasks like liver and lung nodule segmentation.^18^ While DL methods have been extensively applied for brain tissue segmentation in MRI images, their application to CT images remains limited.^19^ Modality transfer between MRI and CT with U‐Net‐based architectures has been used for segmentation of WM and GM, either indirectly, on the synthetic MRI images, or jointly, as a multi‐task model.^20^, ^21^

Given the growing interest in applying DL to medical image analysis, our study aims to build on this foundation. To our knowledge, it is the first attempt to apply deep learning‐based techniques to VMI for brain segmentation. In this initial study, we develop DL models for brain segmentation, using patient images (without pathology) from a dual‐layer CT, to evaluate the feasibility of segmentation and volumetric quantification of the major intracranial tissue (WM, GM, and CSF).

Our code for training and evaluating our models is available at: https://github.com/fwinzell/brainCT.

## 2 MATERIALS AND METHODS

### 2.1 Data collection

The study was retrospectively performed by retrieving images from the picture, archiving and communication system (PACS) at Skåne University Hospital in Lund. The study was approved by the Swedish Ethical Review Authority (dnr 2019–02225) and informed consent was waived.

A pilot study, using two patients for training and one patient for validation, indicated that a sample size of 20 patients would suffice, as model performance showed rapid improvement.^22^

Consecutive noncontrast brain CT (NCCT) examinations between June 2018 and March 2019 on a dual‐layer CT (Philips IQon, Philips Healthcare, Inc, the Netherlands), were considered. Inclusion criteria required patients to be over 18 years of age and to have both a NCCT with spectral data files and a T1‐weighted MRI (from any MR system). A maximum time interval of 2 years between CT and MRI examinations was allowed. Exclusion criteria were pathological findings, metal or motion artifacts, or anatomical changes between the CT and MRI examinations. A senior neuroradiologist (> 20 years of experience) reviewed all cases to ensure they met the criteria. Included patients were randomly assigned to training and test datasets in a 4:1 ratio, resulting in a training set four times larger than the test set.

Using spectral data files, VMI ranging from 40 to 200 keV can be produced using the manufacturer's dedicated software (IntelliSpace Portal 10.1.4.21403, Philips Healthcare Inc, The Netherlands). The attenuation of, and contrast (i.e., difference in attenuation) between, intracranial tissue vary with the VMI level. To provide the models with a broad range of information, three VMIs (50, 70, and 120 keV) were selected. A 50 keV was chosen over 40 keV due to the latter's less favorable noise level. And 70 keV was selected as it most closely resembles the conventional polyenergetic image. Also 120 keV was chosen as attenuation changes become negligible at higher energies.

### 2.2 MR‐based ground truth segmentation

CT and MR images were co‐registered, using a rigid body transformation with SimpleElastix, before the MRI was used to generate ground truth segmentations.^23^ FreeSurfer (Version 7.3.2) was used for an automatic segmentation of brain tissue from MRI.^24^ The quality of the segmentations was assured by a senior neuroradiologist and segmentations with substantial errors were discarded from further analysis.

### 2.3 Model architecture

A simple baseline U‐Net++ model was compared to two U‐Net++ extensions (which we refer to as Aug and Fuse) as well as three U‐Net extensions (Aug, Fuse, and Gated). The baseline was trained using only one VMI and the extensions were designed to leverage spectral information from the three VMIs. The PyTorch‐based framework Monai was used for the baseline model and the base for the extensions.^25^ Instance normalization was used for all models. Parametric rectified linear unit, PReLU, and LeakyReLU were used as activation functions for U‐Net and U‐Net++ models, respectively. The input to the model consists of 2D axial slices of the brain. A pseudo three‐dimensional approach was used, where a number of slices above and below the slice of interest are added as input channels. This provides the model with information on the surroundings, without taking the whole volume into account when segmenting each slice. This approach has been shown to improve performance in MRI brain segmentation, without substantial increases in computational cost.^26^ A cross‐validation procedure was employed on the training data to determine the optimal number of input slices for the pseudo three‐dimensional approach, and to optimize the size of the model (three input slices, 3.5 M for U‐Net, and 2.6 M for U‐Net++), see Table S1 and Figure S1. Additionally, cross‐validation was used to evaluate which VMI to use for the baseline model (70 keV), see Table S2.

**FIGURE 2:**
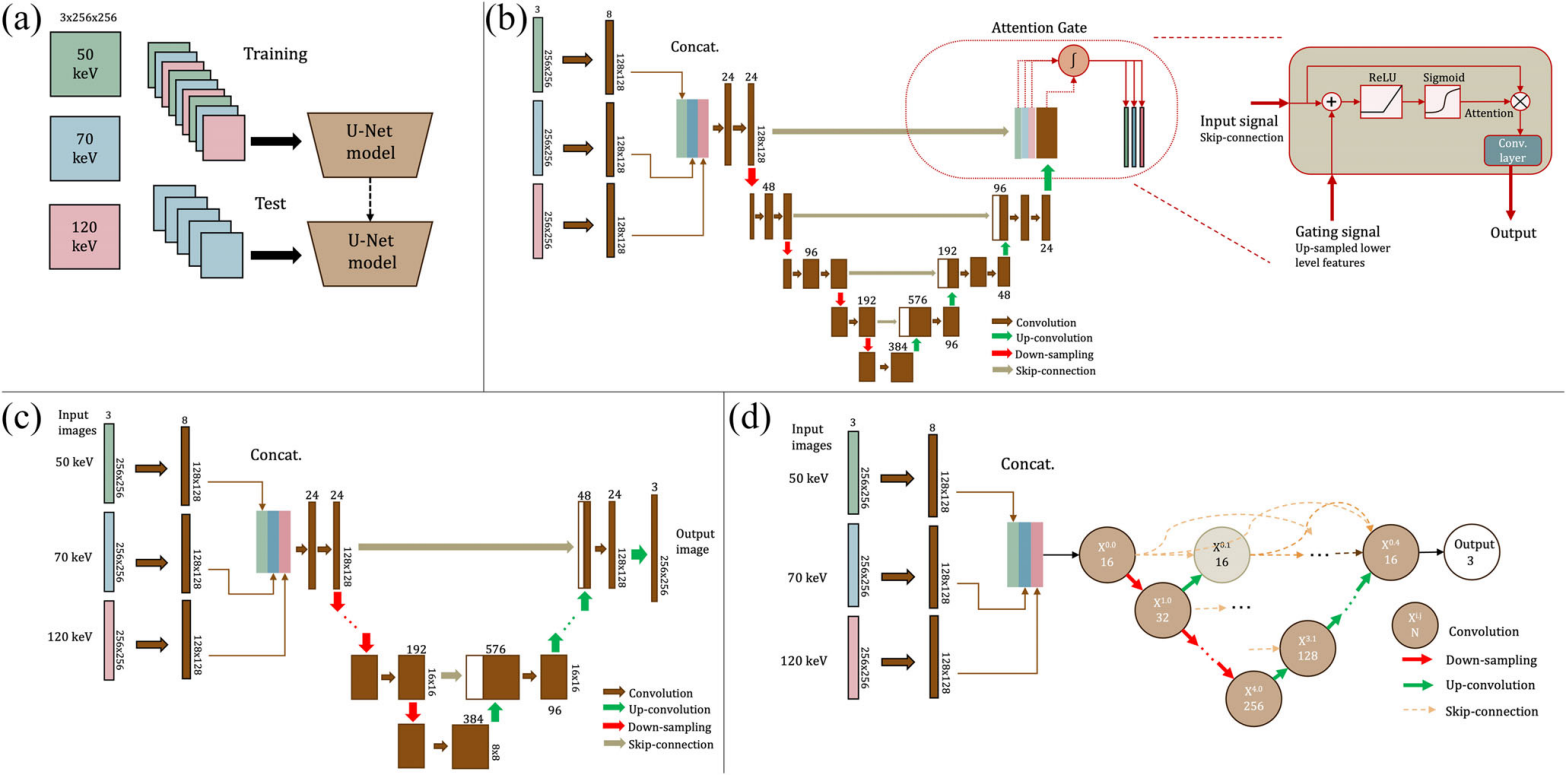
(a) Aug: Using the virtual monoenergetic images (VMI) of 50, 70, and 120 keV as augmentations (i.e. in each epoch, all three VMIs for every patient in the training data are utilized). (b) Gated: The three input pathways with an added attention gate to the output block. Input to the block is scaled with attention weights, computed from the input and gating signals. This enables the network to learn which regions and VMI channels are more important to focus on for each class in the output. (c) U‐Net Fuse and (d) U‐Net++ Fuse: Three separate input pathways, one for each VMI, consisting of one convolutional layer and subsequent activation and batch normalization. The output features from the input layers are then concatenated and sent through the rest of the network.

The first extension, referred to as “Aug” (Figure 2a) was trained using the different VMIs as augmentations, that is, all three VMIs for every patient were used in each epoch. The second extension, referred to as “Fuse” (Figure 2b), involves a novel input layer containing three pathways, one for each of the VMIs (50, 70, and 120 keV). Each pathway, which uses one specific VMI image as input, consists of one convolution layer followed by an activation function before being fused by concatenation and connected to the rest of the model. The third extension, referred to as “Gated” (Figure 2c), builds upon the second by introducing an attention gate between the last up‐convolution and the top skip‐connection, following the 3‐pathway input layer, similar to the Attention U‐Net by Oktay et al.^26^

The models were compared to a generative U‐Net model, referred to as “U‐Net (Gen)” This U‐Net model is extended with an extra decoder, aiming to reconstruct the original MRI, similarly to the approach by Han et al.^21^ Furthermore, the “CTseg” MATLAB algorithm, implemented in the SPM12 MATLAB package, was used for comparison.^15^

### 2.4 Training procedure

A sevenfold cross‐validation was employed for model training. The patients in the training set were randomly divided into seven equally sized folds, and the same folds were used for all models. As the segmentation was performed in a slice‐by‐slice fashion, there was no guarantee that each training batch would include all tissue classes. In the initial stages, it was observed that the models failed to segment CSF when early batches lacked the tissue class. To address this issue, the multi‐class Dice loss function was adapted to compute class‐wise losses and exclude missing classes from contributing to the gradient updates.^27^ All models were trained for 100 epochs using this modified multi‐class Dice loss function.

### 2.5 Performance evaluation

Performance was evaluated using the test set, which had not been used for hyperparameter optimization or model training. The final segmentations were obtained by averaging the outputs from the ensemble of the seven variants, each trained on a different fold, for each model. The variance was estimated using bootstrapping on the test set in a leave‐one‐out manner, resulting in five test iterations, each excluding one patient from the test set. Baseline and augmented (Aug) models were tested on 70 keV images, while the other models were tested using all three VMI levels (50, 70, and 120 keV).

Segmentation accuracy was assessed using Dice similarity coefficient (DSC) and 95% Hausdorff distance (HD). DSC is a spatial overlap metric, ranging from 0 to 1, where 1 represents perfect overlap (ideal segmentation) and 0 indicates no overlap.^28^, ^29^ The HD is defined as the maximum distance between any two nearest points on the borders between the segmentation and ground truth. Hence, a lower value is better. Since this metric is prone to outliers, we used the 95^th^ percentile instead of the maximum. Both metrics were calculated for each segmented 3D volume and presented with 95% confidence interval (CI).

As the developed models (at this stage) are not intended for precision tasks, but rather for volumetric quantification, DSC was chosen as the more important performance metric. Welch's *t*‐test was used to determine if the difference in DSC between the best‐performing model and the other models was significant. The overall significance level was set to 95% (*p* = 0.05) and the estimated p‐values were corrected using Bonferroni correction to adjust for multiple tests by multiplying with seven (i.e., number of compared models). The volumetric accuracy was assessed by calculating the average volume difference (AVD) for each patient and each tissue class:

$$\mathrm{AVD}\left[\%\right]=\frac{V_{GT}^{\left(c\right)}-V_{M}^{\left(c\right)}}{V_{GT}^{\left(c\right)}}\;\cdot 100\%$$

where $V_{GT}^{\left(c\right)}$ and $V_{M}^{\left(c\right)}$ denotes the ground truth volume and the volume estimated by the model respectively for each tissue class, c. Bland–Altman plots were generated by plotting the AVD against the average volume (mean of ground truth and model estimation). To facilitate comparisons between models, the absolute AVD was calculated and averaged for each tissue class.

## 3 RESULTS

### 3.1 Patient summary

A total of 1474 patients were initially considered for inclusion. From this pool, 75 patients were identified with both a NCCT with spectral data files and a T1‐weighted MRI. About 49 patients were excluded (36 due to pathology, 5 due to discrepancies between CT and MR, 3 due to artefacts, and 5 due to poor‐quality ground truth segmentations) resulting in a final cohort of 26 patients (Table 1).

**TABLE 1:** Summary of patient characteristics and CT parameters (scan and image reconstruction) for the training/validation set and the test set.

		Training/validation	Test
Patient characteristics	Number of patients	21	5
	Men/women (%/%)	5/16 (24%/76%)	0/5 (0%/100%)
	Median age (IQR^a^) [years]	67 (53–75)	66 (56–75)
CT parameters	Tube voltage [kV]	120	
	Collimation [mm]	40	
	Pitch	0.359	
	Rotation time [s]	0.33	
	Reconstruction method and kernel	iDose level 1, UB	
	Reconstructed slice thickness [mm]	1	
	Median CTDI_vol_ ^b^ (IQR) [mGy]	38 (36–43)	41 (36–42)

^a^ IQR = Inter‐quartile range, ^b^CTDI_vol_ = Volumetric computed tomography dose index.

### 3.2 Final models

The two augmented models, U‐Net++ (Aug) and U‐Net (Aug), performed best in terms of DSC across all tissue classes (WM 0.84 (CI 0.81–0.86), GM 0.77 (CI 0.76–0.79), CSF 0.88 (CI 0.84–0.92)), compared to all other models (Table 2). Although performance was equal regarding WM and GM segmentation, U‐Net++ (Aug) was slightly superior regarding CSF segmentation. The difference in segmentation accuracy between the best performing model, U‐Net++ (Aug) and all other models was evaluated using Welch's *t*‐test (Table 2). DSC was significantly higher for WM and GM compared to U‐ Net++ (Baseline), as well as U‐Net (Gen), and CTseg, U‐Net++ (Aug) had significantly higher DSC for all tissue classes compared to U‐Net ++ (Fuse). For all other models, the segmentation accuracy for CSF was not significantly different. There were no significant differences between U‐Net++ (Aug) and the second‐best performing U‐Net (Aug).

**TABLE 2:** Model performance for each tissue class (gray matter (GM), white matter (WM), and cerebrospinal fluid (CSF)), expressed as the overlap between model and ground truth segmentation using the Dice Score Coefficient (DSC), 95% Hausdorff distance (HD), and the absolute average volume difference (AVD) ± standard deviation. Standard deviations are a result of bootstrapping the test set in a leave‐one‐out fashion. The highest DSC, lowest HD, and lowest AVD, respectively, are indicated in bold for each tissue class. *p*‐values are comparing the Dice Score Coefficient (DSC) for each tissue class between the best performing model (U‐Net++ (Aug)) and all other models. Significance level was defined as *p* = 0.05 and significant *p*‐values are indicated in bold and by an asterisk (*).

		Segmentation accuracy			*p*‐values			Absolute average volume difference [%]		
Model		WM	GM	CSF	WM	GM	CSF	WM	GM	CSF
U‐Net++ Baseline	DSC	0.81 (0.79–0.84)	0.75 (0.73–0.77)	0.86 (0.81–0.91)	<0.01*	<0.01*	0.61	14 (5–23)	22 (16–28)	9 (4–14)
	HD [mm]	3.1 (2.9–3.3)	2.4 (2.3–2.5)	2.4 (2.0–2.8)	–	–	–			
U‐Net++ Fuse	DSC	0.82 (0.80–0.84)	0.76 (0.74–0.78)	0.83 (0.78–0.89)	0.02*	0.03*	<0.01*	10 (4–17)	21 (16–28)	7 (4–9)
	HD [mm]	3.0 (2.8–3.1)	2.4 (2.3–2.5)	20.2 (9.4–31.0)	–	–	–			
U‐Net++Aug	DSC	**0.84 (0.82–0.86)**	**0.77 (0.76–0.79)**	**0.88 (0.84–0.92)**	NA^a^	NA	NA	10 (3–18)	25 (17–32)	**7** **(4–9)**
	HD [mm]	2.9 (2.6–3.1)	2.4 (2.2–2.7)	**2.0 (1.9–2.2)**	–	–	–			
U‐Net Aug	DSC	0.84 (0.82–0.85)	0.77 (0.75–0.79)	0.87 (0.83–0.92)	4.41	1.05	4.13	8 (2–15)	30 (24–37)	8 (6–10)
	HD [mm]	**2.7 (2.4–3.1)**	2.4 (2.2–2.6)	**2.0 (1.8–2.3)**	–	–	–			
U‐Net Fuse	DSC	0.83 (0.81–0.85)	0.76 (0.74–0.78)	0.85 (0.80–0.91)	0.07	<0.01*	0.18	9 (3–15)	21 (14–28)	11 (5–18)
	HD [mm]	3.0 (2.8–3.1)	**2.3 (2.2–2.4)**	4.9 (0.1–9.9)	–	–	–			
U‐Net Gated	DSC	0.83 (0.81–0.84)	0.76 (0.74–0.78)	0.86 (0.80–0.91)	0.06	0.01*	0.32	**7** **(2–12)**	23 (16–29)	9 (5–12)
	HD [mm]	3.0 (2.8–3.1)	2.4 (2.3–2.5)	4.7 (0.2–9.2)	–	–	‐			
U‐Net Generative	DSC	0.81 (0.77–0.84)	0.75 (0.73–0.77)	0.86 (0.81–0.91)	<0.01*	<0.01*	0.98	32 (2–44)	18 (13–22)	11 (4–18)
	HD [mm]	3.5 (3.0–4.0)	2.5 (2.3–2.7)	2.2 (1.9–2.6)	–	–	–			
CTseg	DSC	0.79 (0.78–0.80)	0.70 (0.69–0.72)	–	<0.01*	<0.01*		22 (19–26)	**13** **(6–21)**	–
	HD [mm]	3.2 (3.2–3.3)	2.7 (2.4–2.9)	–	–	–	–			

^a^ NA = Not Applicable.

95% HD varied from 2.3 to 3.9 mm for GM and WM. The variation in CSF was larger, ranging from 2.0–20.2 mm. The lowest 95% HD was found for U‐Net (Aug), U‐Net (Fuse), and U‐Net++ (Aug) for WM, GM, and CSF, respectively.

### 3.3 Volume estimation

**FIGURE 3:**
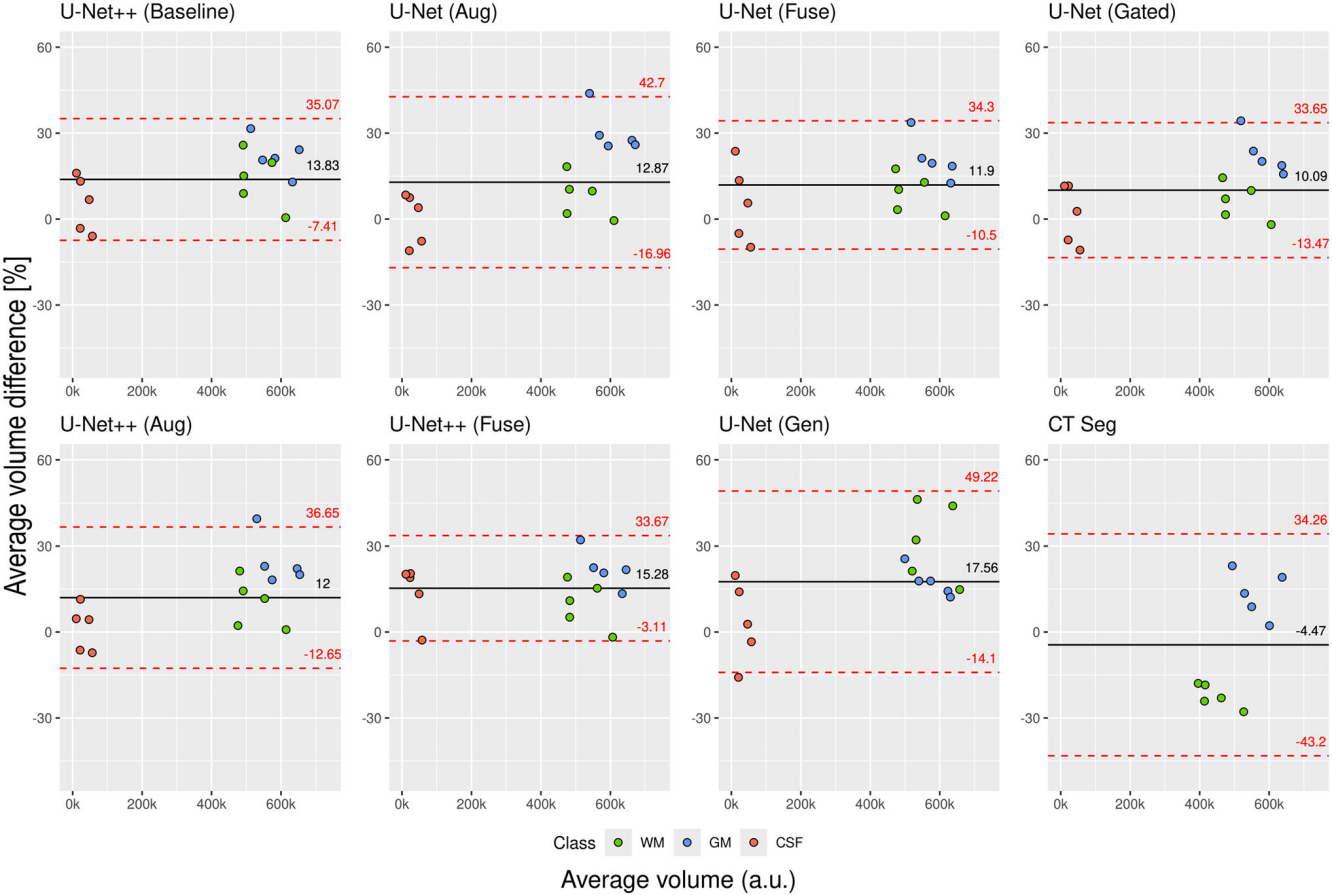
Bland–Altman plots for the average volume difference for all test cases, all tissue classes and all models, compared to the ground truth segmentation. The *x*‐axis presents the mean value of the calculated and ground truth volume. The average error, across all cases and classes, for each model is indicated by a solid black line. a.u. = arbitrary units, CSF = Cerebrospinal fluid, GM = Gray matter, WM = White matter.

AVDs for all models were of the same order of magnitude for each intracranial tissue (Figure 3, Table 2). Except for CTseg, all models on average overestimated the total volume for all tissue classes. GM showed a tendency for larger overestimations, while errors in CSF were generally lower and distributed across both positive and negative percentage differences. U‐Net (Gated), CTseg, and U‐Net++ (Aug) achieved the lowest absolute AVD for WM, GM, and CSF, respectively (Table 2).

U‐Net (Gated) produced, averaged across all tissue classes, the most accurate volumetric estimation. (Figure 3). CTseg, although not including the CSF class, achieved the lowest AVD. However, this is caused by systematic overestimation of GM and underestimation of WM volume.

### 3.4 Visual evaluation

**FIGURE 4:**
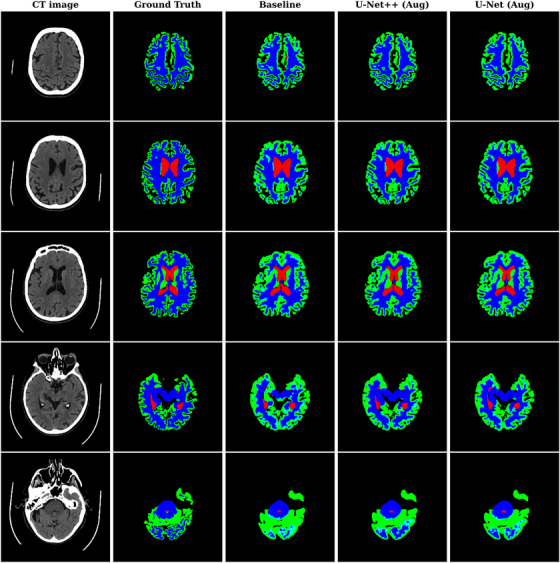
Segmentation results for a patient in the test set. From left to right: CT image, MR‐based ground truth, baseline U‐Net++, best performing U‐Net++ (Aug), and U‐Net (Aug). White matter—green, Gray matter—blue, Cerebrospinal fluid—red, and Background—black.

The ground truth segmentation for one of the test cases presented side‐by‐side the baseline and best performing U‐Net++ (Aug) and U‐Net (Aug) segmentations (Figure 4). It was visually confirmed that all models lack the high resolution of the ground truth, which is most apparent in the more inferior regions of the cerebrum and the cerebellum, resulting in an overestimation in total volume for both GM and WM.

## 4 DISCUSSION

This study explored the feasibility of DL segmentation of WM, GM, and CSF using VMI obtained from a dual‐layer CT. We developed and compared the performance from a baseline model to six other models. The baseline model differed from the other models in that it utilized only one VMI level (70 keV), while all other models in different ways used all three VMI levels (50, 70, and 120 keV). Model optimization showed that VMI of 70 keV was optimal for the baseline model, likely as a result of 70 keV providing the best balance between tissue resolution and noise.

Our best performing model, U‐Net++ (Aug), achieved DSC of 0.84 (CI 0.81–0.86), 0.77 (CI 0.76–0.79) and 0.88 (CI 0.84–0.92) for WM, GM, and CSF, respectively. The baseline model performed worst for WM and GM, with DSC of 0.81 (CI 0.79–0.84) and 0.75 (CI 0.73–0.77). The difference was significant and could be explained by the models achieving an improved discrimination between tissue classes with multiple VMIs. The second‐best performing model was U‐Net (Aug), suggesting that to believe that the increased amount of training data provided by the augmented input approach could be an important contributing factor. The ability to generate multiple VMIs, which can act as a form of augmentation and enhance segmentation performance, could be seen as an advantage of dual energy CT in the context of DL development. Although other models out‐performed U‐Net++ (Aug) in HD for WM and GM, HD remained low at 2.9 (CI 2.6–3.1) mm for WM, 2.4 (CI 2.2–2.7) mm for GM, and 2.0 (CI 1.9–2.2) mm for CSF. HD was generally low across models (median HD < 4.0 mm), with the exception of CSF for the more complex (Fuse) and (Gated) architectures; the largest outlier was U‐Net++ (Fuse), with an 20.2 (CI 9.4–31.0) mm. CSF forms thin and narrow structures, which makes distance‐based metrics such as HD sensitive to small local deviations. Minor differences in delineation can increase HD without substantially affecting DSC. In our results, both the (Fuse) and (Gated) models showed higher HD for CSF, likely reflecting small local variations introduced by how these architectures combine information. In contrast, the (Aug) approach appeared more robust, possibly because all VMIs are incorporated in a more uniform way, leading to a more consistent result.

The segmentation performance of our proposed model was at par with previous studies on MRI‐based deep learning segmentation. In these studies, DSC has been reported in the range of 0.80–0.96 depending on patient cohort (adults or infants) and structure at interest.^30^, ^31^, ^32^ However, MRI‐based deep learning models for a simple segmentation of WM and GM are still superior to our model. Our proposed model showed significantly better performance than CTseg, which could be explained by CTseg being based on a different set of CT images, while our models were trained specifically using VMIs from this CT scanner.

Volume estimation should be interpreted carefully, as volumetric accuracy does not entail spatial accuracy. Thus, overlap metrics are the superior choice for performance evaluation. However, as the estimation of brain tissue volume is highly relevant for diagnosis and monitoring of several neurodegenerative diseases, we believe that it should be considered even in a feasibility study. Our models systematically overestimated both WM and GM volume. The U‐Net++ (Aug) had an absolute average error of 13% (WM 10 (CI 3–18)%, GM 25 (CI 17–32)%, CSF 7 (3–9)%). Meanwhile, the best performing models, U‐Net (Gated) and (Fuse), had an absolute average error of 12%. But, for all models, errors larger than 30% were found for individual cases. The difference in intracranial tissue volume, between disease afflicted and healthy controls will vary with disease, structure of interest and measurement method. Reported differences for Huntington's disease has been observed to vary in the range of 5%–30%.^33^, ^34^ Similarly, volumetric differences in structures between mild cognitive impairment and dementia can vary from 5%–40%.^7^ Given the average error of 10%–12%, our best performing models may need further refinement to be clinically relevant, but is an important step in the right direction.

Visual inspection of the final segmentations (Figure 4) confirms that none of the models delineates fissures with the same resolution as MR‐based segmentation, explaining the overestimation of WM and GM. Performance was notably poor in the cerebellum. The cause may be the limited spatial and contrast resolution, as well as limitations in the ground truth segmentation. Development of segmentation methods is challenged by the availability of a reliable ground truth. In this study, the ground truth was generated using MRIs and FreeSurfer. However, FreeSurfer struggled to accurately delineate the cerebellum, suggesting that manual segmentation, while time‐consuming, may be necessary to achieve more accurate results in this region.

This study has several limitations. The dataset consisted of only 26 patients without pathology, which was considered sufficient for a feasibility study and given the focus on a single CT system. Future work should validate our proposed model on patients with pathologies and across multiple CT systems and imaging conditions, which will require a much larger dataset. HU values in VMI tend to be more consistent across CT systems compared to conventional polyenergetic images, which may enhance the model's generalizability. Due to the lack of publicly available CT datasets with VMIs, comparisons with other data or models were not performed in this study. To enhance performance and user usability, the deep learning model's output should include a confidence estimate or probability of accuracy, providing greater transparency and aiding in interpretation. Furthermore, the performance of our models is dependent on the performance of FreeSurfer. During data curation, certain cases were excluded due to inaccurate ground truth segmentation, which could bias our results.

While CT‐based segmentation cannot yet match the precision of MRI‐based methods, the progress shown in this study suggests that it, with refinement, may achieve clinically relevant accuracies. Improvements in CT image quality could further enhance the segmentation accuracy, such as using photon counting CT images which offer lower noise levels and improved spectral and spatial resolution. With further advancements in image reconstruction and deep learning models, the gap may continue to narrow. Achieving reliable segmentation from CT images would offer substantial benefits, allowing for quantitative analysis in situations where MRI is unavailable or contraindicated.

## 5 CONCLUSIONS

This study demonstrates the feasibility of dual layer CT‐based segmentation and volumetric estimation of intracranial tissues using DL and VMIs. The segmentation accuracy of the best‐performing model, U‐Net++ (Aug), was high with DSC of 0.84, 0.77, and 0.88 for WM, GM, and CSF, respectively, and an average volumetric error of 13%. The U‐Net++ (Aug) model was significantly improved compared to the baseline model, suggesting that the addition of spectral information can enhance model performance.

## CONFLICT OF INTEREST STATEMENT

AT has received research funding from Siemens Healthineers. JW is a founder and shareholder of Uman Sense AB and has been a consultant speaker from Siemens Healthineers, Balt group and Medtronic Inc.

## Supporting information



Supporting Information

Supporting Information

Supporting Information

Supporting Information
