# Supplementary material for: Dual energy CT and deep learning for an automated volumetric segmentation of the major intracranial tissues: Feasibility and initial findings
Source: Med Phys. 2025 Dec 21;53(1):e70217. doi: 10.1002/mp.70217 (PMC12719377; doi:10.1002/mp.70217)
Supplement: Supplementary file 1 — Supporting Information [file MP-53-0-s001.docx]

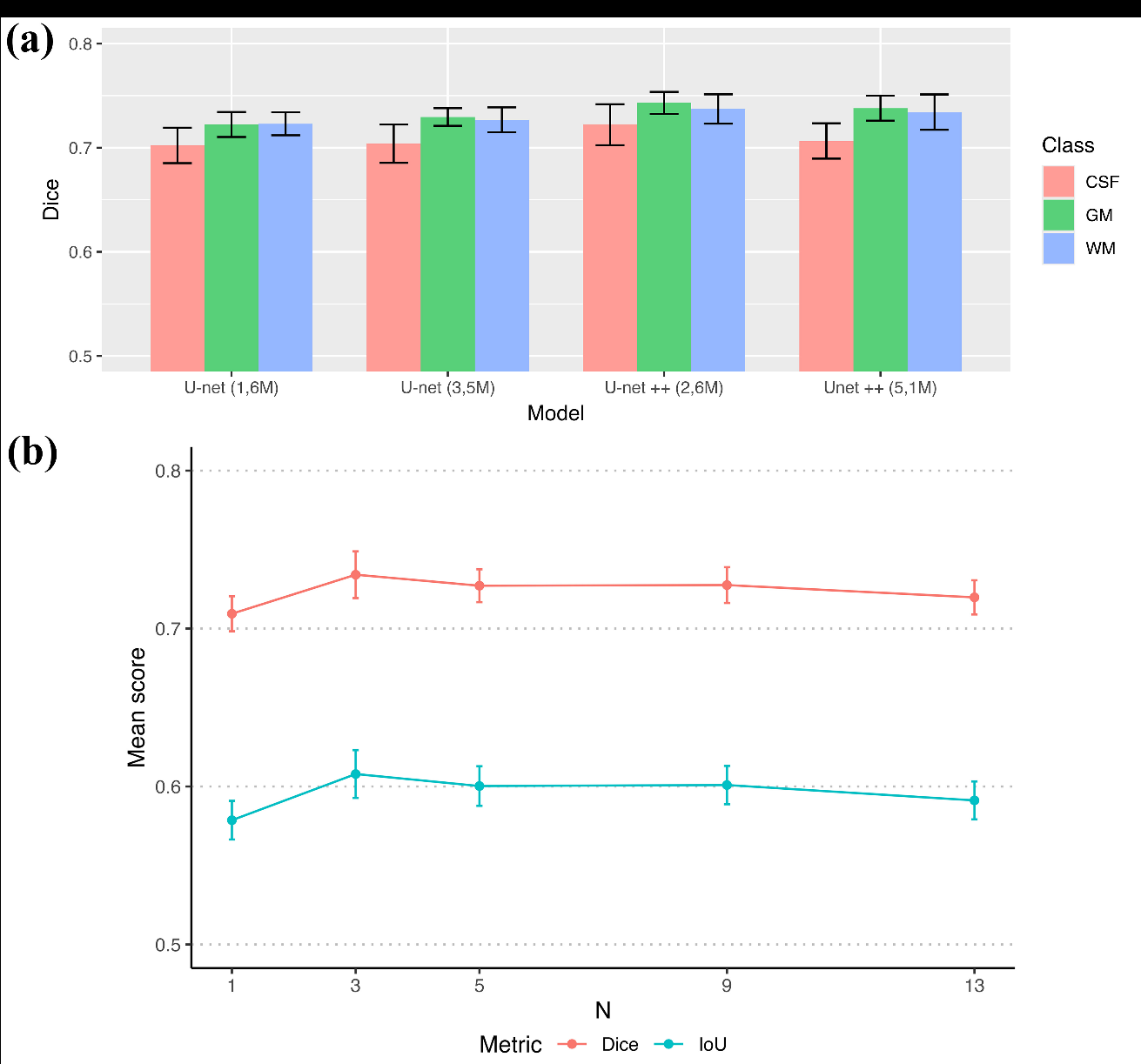


**Figure S1.** **a)** Comparison of two U-Net and two U-Net++ models with a varying number of parameters using a 6-fold cross validation setup. **b)** Effect of the number of input slices (N) on a U-Net++ (2.6M) model from 1 to 13. Error bars indicate the standard deviation following a 6-fold cross-validation procedure.
