# Supplementary material for: Dual energy CT and deep learning for an automated volumetric segmentation of the major intracranial tissues: Feasibility and initial findings
Source: Med Phys. 2025 Dec 21;53(1):e70217. doi: 10.1002/mp.70217 (PMC12719377; doi:10.1002/mp.70217)
Supplement: Supplementary file 2 — Supporting Information [file MP-53-0-s003.docx]

| **Model architecture** | **DSC** | | |
| --- | --- | --- | --- |
|  | **WM** | **GM** | **CSF** |
| U-Net (1.6M) | 0.723 ± 0.01 | 0.722 ± 0.01 | 0.702 ± 0.02 |
| **U-Net (3.5M)** | **0.727 ± 0.01** | **0.730 ± 0.01** | **0.704 ± 0.02** |
| **U-Net++ (2.6M)** | **0.737 ± 0.01** | **0.743 ± 0.01** | **0.722 ± 0.02** |
| U-Net++ (5.1M) | 0.734 ± 0.02 | 0.738 ± 0.01 | 0.707 ± 0.02 |
