# Supplementary material for: Dual energy CT and deep learning for an automated volumetric segmentation of the major intracranial tissues: Feasibility and initial findings
Source: Med Phys. 2025 Dec 21;53(1):e70217. doi: 10.1002/mp.70217 (PMC12719377; doi:10.1002/mp.70217)
Supplement: Supplementary file 3 — Supporting Information [file MP-53-0-s004.docx]

| **Model architecture** | **VMI** | **DSC** | | |
| --- | --- | --- | --- | --- |
|  |  | **WM** | **GM** | **CSF** |
| U-Net++ | 50 keV | 0.715 ± 0.01 | 0.717 ± 0.01 | **0.629** ± 0.07 |
| U-Net++ | 70 keV | **0.721** ± 0.01 | **0.719** ± 0.01 | 0.621 ± 0.10 |
| U-Net++ | 120 keV | 0.705 ± 0.01 | 0.711 ± 0.01 | 0.577 ± 0.09 |
