# Supplementary material for: Dual energy CT and deep learning for an automated volumetric segmentation of the major intracranial tissues: Feasibility and initial findings
Source: Med Phys. 2025 Dec 21;53(1):e70217. doi: 10.1002/mp.70217 (PMC12719377; doi:10.1002/mp.70217)
Supplement: Supplementary file 4 — Supporting Information [file MP-53-0-s002.docx]

| **Model architecture** | **Method** | **Average time [s]** |
| --- | --- | --- |
| **U-Net++** | Baseline | 113 ± 8 |
|  | Fuse | 100 ± 6 |
|  | **Aug** | 112 ± 6 |
| **U-Net** | Aug | 13 ± 1 |
|  | Fuse | 13 ± 1 |
|  | Gated | 15 ± 1 |
| **U-Net** | Generative | 30 ± 2 |
